# Development and evaluation of a low-cost database solution for the Community Paramedicine at Clinic (CP@clinic) database

**DOI:** 10.1371/journal.pdig.0000689

**Published:** 2024-12-27

**Authors:** Ricardo Angeles, Krzysztof Adamczyk, Francine Marzanek, Melissa Pirrie, Mikayla Plishka, Gina Agarwal

**Affiliations:** Department of Family Medicine, McMaster University, Ontario, Canada; Fundación Progreso y Salud: Junta de Andalucia Consejeria de Salud y Familias Fundacion Progreso y Salud, SPAIN

## Abstract

The Community Paramedicine at Clinic (CP@clinic) program is a community program that utilizes community paramedics to support older adults in assessing their risk factors, managing their chronic conditions, and linking them to community resources. The aim of this project is to design a low-cost, portable, secure, user-friendly database for CP@clinic sessions and pilot test the database with paramedics and older adult volunteers. The CP@clinic program database using the Microsoft Access software was first developed through consultation with the CP@clinic research team. Next, the database was pilot tested with two sets of older adults and one set of paramedics to assess user experience. Volunteers completed a survey regarding their perceptions of the level of difficulty when using the database. A computer-based database was the best option as it provided flexibility while reducing costs. The final database should perform calculations and summarize risk assessment data, provide recommended resources, generate automated reports, capture changes in medical and medication history, and ensure that the sensitive information is secure. During pilot testing, the older adult participants and the paramedics indicated that the database was easy to use. This low-cost, user-friendly and secure database captures initial and follow-up data, incorporates algorithms that guide the paramedics, and calculates risk factor scores for the participants. This solution to a healthcare database is translatable to other health research studies in which ongoing patient data is collected electronically and longitudinally.

## Author summary

We have previously developed and launched a community health and wellness clinic called the Community Paramedicine at Clinic (CP@clinic) Program, which utilizes community paramedics to deliver basic health and risk factor assessments to older adults in the common areas of their residence buildings. This program provides older adults with health education and links them to relevant community resources. In order to accurately keep track of the demographic information, health assessments, and referrals for each of the participants, we needed to develop a secure database that met the needs of the CP@clinic program. Ideal features of the database were that it should be portable, user-friendly and low-cost. In our study, we invited both paramedics and older adult volunteers to assist with the development of a CP@clinic program database using the Microsoft Access software. Our expert panel came up with the requirements for the database and the paramedics and older adult volunteers provided feedback on the functionality and friendliness of the user interface. Through our consultations with the expert panel and from the experiences of our volunteers, we successfully developed a database that met all of our requirements. Our study provides insight into the methods used to develop a cost-effective, highly secure, portable, and easy-to-use database that would be of interest to healthcare researchers that are collecting longitudinal data electronically.

## Introduction

Older adults living in social housing experience poorer health conditions, have challenges navigating the health system, and are often unaware of their health risks [1,2]. The Community Paramedicine at Clinic Program (CP@clinic) is a low-cost community program that was developed to assess community dwelling older adults and empower them to identify behaviour risk factors that may impact their health and wellbeing. The program supports older adults in managing these risk factors to prevent emergency health situations, link them with community resources, and communicate their health information to their primary care physician. This coordination in health care services results in better health outcomes leading to less adverse health issues and fewer EMS calls [3].

The CP@clinic program has been refined through evidence-based evaluations [3–5], experiential program implementation, and quality improvement activities. CP@clinic assesses individuals’ blood pressure, diabetes risk, and falls risk [3,4]. The program is delivered in common areas of subsidized seniors’ residence buildings where older adults are guided through a defined risk assessment process by trained community paramedics. The paramedic then provides relevant health education and referrals to resources and the assessment results are faxed to the participants’ family physicians. Demographic information, risk assessment data, risk factor discussions, and referrals are securely entered and stored in the CP@clinic database on a laptop computer. Previous research has indicated that participants in the program experienced timely access to health information and services, support for personal health goals, better understanding of the healthcare system and they felt a stronger sense of community [6]. Consequently, this program holds significant advantages for the community.

There is a critical need for a secure, inexpensive, portable, and user-friendly database to support the CP@clinic program. This study addresses the gap in literature by developing a database solution tailored to the unique requirements of the CP@clinic program, operating in subsidized seniors’ residences. By conducting risk assessments for various health factors and securely storing and transmitting data to family physicians, it aims to overcome challenges of mobility, technology access, and resource limitations. The research contributes valuable insights to healthcare researchers, offering a model for managing health data in community-based programs for older adults in similar settings. The overall objective of this study was to design and pilot test a project database that meets specific criteria: being low-cost, portable, secure, user-friendly, and possessing the necessary capabilities to facilitate the implementation of the CP@clinic program. Insights gained from participants in Phases One and Two were used to enhance the database. [3,4]. This research ensures that the CP@clinic program can effectively collect, store, and utilize data to assess participants’ health risks and outcomes, ultimately contributing to improved healthcare delivery in community-based settings.

## Materials and methods

### Research design

This study was conducted in two phases carried out in Ontario, Canada. In Phase One, database requirements were gathered from consultations with an expert panel and through a review of the CP@clinic protocol, algorithms and assessment tools to determine the database platform, functionality, contents, and security required to meet the needs of CP@clinic. In Phase Two, utility testing was conducted with potential database users to ensure a user-friendly interface. A cyclical iterative design process was utilized where knowledge gained from one iteration was used to inform the next iteration [7,8]. Since it was not possible to know upfront what the final database should look like, an approach was used that allowed multiple product refinements. The study was approved by the Hamilton Integrated Research Ethics Board (HiREB).

### Participants

Expert Panel: The CP@clinic research team participated in this study as an expert panel. The multidisciplinary research team consists of a family doctor with over 30 years of experience using electronic medical records, a public health nurse with over 30 years of experience using medical records, a paramedic with over 15 years of experience with record keeping for acute emergency medical services, a research associate with over 25 years of experience in database and statistical programs, a program coordinator, and an Information Technology (IT) manager with over 20 years of experience maintaining and hosting electronic medical records and networking.

Potential Database Users: Although the current CP@clinic model had paramedics staffing the program, there was potential for a volunteer peer-led, paramedic-supervised model in the future; therefore, it was important to consider older adults (55 years and older) as potential users when designing the database. A purposive sample of five paramedics and ten older adults who shared similar characteristics with potential volunteers were recruited to test the database usability. All participants were over the age of 18. We recruited 5 paramedics and 10 older adults to allow us to conduct the iterative process of developing, revising, and improving the database. Three paramedics participated in the initial development of the database, 5 older adults participated in the initial testing, 5 older adults participated in repeat testing after revisions were incorporated; 2 of the paramedics involved in the initial development and 2 new paramedics were involved in the final testing.

### Data gathering and testing procedures

To efficiently implement CP@clinic, a project-specific smart database was required with the capability to:

- Capture initial and follow-up data
- Guide the paramedic personnel in administering the risk assessments survey
- Calculate and summarize the participants’ risk factor scores
- Direct individuals in different predetermined clinical algorithm pathways according to their risk levels and evidence-based medical directives
- Provide information about local resources available to manage risk factors that are tailored to the participants’ situation
- Provide automated alerts based on specified evidence-based algorithms to facilitate ongoing data capture (e.g. 6 month follow-up for fasting blood glucose)
- Generate automated reports for researchers, participants, and family physicians
- Securely sync data from project sites to a central, project-specific server

In Phase One, the expert panel reviewed the contents, platform, functionality, and data security elements required for the database. Examining the CP@clinic protocol, the database should include participant demographic information, physician contact information (including a stored physician list), physical measurements (e.g. height, fasting blood glucose), medical and medication history, a resource list, and a record of risk factor discussions. It also needed to include publicly available risk assessment tools that identified risk factors among older adults. For the platform, functionality, and data security elements of the database, the decision to choose specific features was guided by the following principles: feasibility of implementing, cost of implementation, and possibility of scaling-up the technology.

Different questionnaires were evaluated by the expert panel to determine the contents of the database. The panel determined whether the tools met the following criteria: tested for reliability and validity in an older adult population, appropriate for the subsidized housing setting, could be delivered by volunteers, and feasible to be completed in a drop-in clinic setting. The different elements of the database (risk assessment tools and processes) were evaluated during weekly panel meetings for one month until all members came to a consensus on the features to be included in the initial database. The consensus building process involved evaluating the different tools against criteria, incorporating relevant ones into the database flow, then reviewing the database flow and making appropriate adjustments. This was repeated every week until consensus was established.

In Phase Two, the database was pilot tested with two mutually exclusive sets of older adults (five per set) and one set of paramedics. The pilot tests were done in different settings that were convenient to the participants. The settings included a research office building, the participants’ home, or a common space area in an apartment building. To evaluate the usability of the database, user experience was assessed. The variables studied included the following user-experience metrics: difficulty navigating the database, time to complete the database forms, and a prediction of whether paramedics and older adult volunteers will be able to complete the database forms. Participants were asked to enter their own health-related information or fictional information to complete the forms in the database. They subsequently completed a survey with 5-point Likert scale responses regarding their perceptions of the level of difficulty when using the database, the time needed to complete the forms, whether they thought paramedics or volunteer older adults would be able to complete this task, their familiarity with computers, their age, and any suggested changes to render the CP@clinic database more user-friendly. The questionnaire was developed to fit the purpose and elements of the database. This content was validated by the Expert panel. The results for each round of pilot testing were used to improve the database, which was then tested using the next group of participants.

### Analysis

Data from the Phase Two survey was summarized using frequency distribution. The aim of the analysis was to evaluate the perceptions of older adults and paramedics regarding how user-friendly the database was.

## Results

### Phase one: Database requirements and development

Through consultations with the expert panel and a review of the program documents, a set of database requirements were gathered and decisions were made regarding the database contents, platform, functionality, and data security. For each database element below, the identified requirements have been listed, followed by the solutions implemented.

### Contents

The initial set of questionnaires presented to the expert panel included tools from the Canadian Community Health Survey (CCHS) [9], the Canadian Hypertension and Education Program (CHEP) [10], and the Canadian Diabetes Risk (CANRISK) questionnaire. Furthermore, since older adults living in subsidized housing had poor health status [1] and have frequently called 911 due to falls [11], health related quality of life questionnaires [12,13] (Euroqol 5 dimensions 3 levels) [14] and falls assessment (Timed-up-and-go or TUG test) [15] were added. The expert panel’s consensus of tools to be included in the database were as follows:

- Selected demographic variables (age, sex, ethnicity, marital status) and risk factor questions that were similar to what was collected in the CCHS (representative of the general population) so that comparisons can be made
- The CANRISK questionnaire as this was the only validated tool assessing diabetes risk in Canada
- Euroqol 5 dimensions 3 levels (EQ5D 3L) as this was the shortest tool for quality of life assessment and was used by health economists in Canada
- The TUG test since this was the standard tool recommended for falls assessment in older adults

### Platform

The expert panel identified the need for the optimal database platform to be low-cost, secure, flexible (not dependent on internet access), capable of implementing complex algorithms and computations, and easy to use. The advantages and disadvantages for each of the database platforms considered are summarized in Table 1.

**Table 1:** Advantages and Disadvantages of different database platforms.

Database Platform	Advantages	Disadvantages
Web-based database/ Web-based (Tablet/iPad) Applications	• Data is stored in real time on a secure server • No data is stored in the device, therefore no security issues if the device is lost • Best choice for a secure database • Works on any device (iPad, Tablet, Chromebook, or laptop) with an internet browser	• Always requires internet connectivity for database to be available • Requires IT specialist development and ongoing maintenance support (more expensive option)
On-board Tablet/iPad Applications	• Can be used off-line and syncs when Wi-Fi is available • May be easier to develop compared to web-based database • More flexible to future database changes	• Data is kept in the device, reducing data security issues • May be less user-friendly for people who prefer typing into a physical keyboard • Requires a developer, which can be expensive
Computer-based database (Windows-based)	• Easy to develop, does not need IT specialist support • Very flexible. Data can be stored in a network folder and can be as secure as a web-based portal or on-the computer to be synced to a network folder when Wi-Fi is available • Database itself is usually free of charge	• Mainly for laptops • Data is kept in the device–data security issues

Accordingly, the computer-based database was selected since it was more flexible and less expensive, which was important for the sustainability of the CP@clinic program. This option did not require internet connections and gave researchers the ability to modify the database quickly.

### Functionality

The database needed to be capable of multiple functions to support the efficient and effective implementation of CP@clinic. The database needed to capture initial and follow-up data entered by a user, perform calculations and summarize information to provide a risk assessment score, provide alerts to the user based on the results, compute participant age from the date of birth, the body mass index from the weight and height, and the CANRISK score, and to summarize all the participants’ risk factors, while guiding the user through the assessment process as they complete the forms and the risk assessment tools. Furthermore, once the participant’s risk was assessed, the database needed to provide resources available to manage risk factors that are tailored to the participant’s situation and generate automated reports for the researchers, participants, and the participants’ family physicians. To meet these database needs, the final format of the CP@clinic database initially used free software from the Centres for Disease Control, Epi Info 7 [16] but switched to Microsoft Access 2010 [17]. The database was structured like an electronic medical record, allowing cumulative information by participant.

### Data security

The security of the data was a priority since the data contained personal health information. The database needed to be secure at the level of user access, the storage device (in case of theft or loss of the laptop), and data transmission (the data was transmitted to a secure project server). The CP@clinic data collected during sessions was locally stored in the CP@clinic laptop instead of directly in the CP@clinic central server to minimize the issue of poor internet connection in some locations. Moreover, the CP@clinic database is located in a hidden encrypted container/folder in the laptop using the Safehouse software [18]. The container and the database are password protected with a 20 character random alphanumeric password that was saved in a specialized Yubikey [19]. After each CP@clinic session, the paramedics return to the Hamilton Paramedic Service home base and are instructed to connect the laptop to their internal internet and synchronize the database to the CP@clinic central server using the FreeFileSync software [20]. The network folder on the central server saves the updated version of the CP@clinic database and maintains previous versions of the CP@clinic database. The CP@clinic central server is an SME Linux enterprise server, based on the CentOS operating system 7.5, that allows secure file sharing for authorized users [21]. The server is located on a secure university network, behind a university firewall, and is accessible via a virtual private network (VPN) connection for remote clients. The server is supported with nightly back-up onto a secondary onsite server with the same specifications and a third level secure off-site server backup. Access to the three servers is limited to approved individuals. In addition, users were unable to open unsafe web pages that could compromise the integrity of the operating software and all applications were locked one minute after the laptop was left unplugged and unused or when the laptop was in sleep mode. Lastly, anti-theft software, “Prey” [22], was installed to delete all files and take a snapshot of the person using the computer once the laptop was registered as stolen. All the software used in setting up the laptop system were freeware except for the anti-theft software, which had a minimal fee.

### Phase two: Pilot testing the user-experience

#### Initial development and first pilot test

The initial database using Epi Info 7 was pilot-tested with three paramedics who found the database processing speed to be slow, despite using a laptop with high-end specifications. They also perceived the UI to not be user-friendly since it had a different appearance than commonly used Microsoft (MS) programs. Since the CP@clinic program had different paramedics managing the program, and potentially older adult volunteers, the database needed to be simplified to accommodate users with varying levels of experience with technology. The database was switched to MS Access 2010 (full version and the free Runtime version) [17] because it was hypothesized to have a more instinctive appearance and functionality. Resultantly, the paramedics found the UI familiar and the database simple to use. The CP@clinic database was rebuilt and enhanced using this platform.

#### Pilot test with older adults (set 1)

During the first pilot test, all five older adults found the database easy to complete and agreed that volunteer older adults would be able to complete the database. Although their experience with the database was generally positive, three of them preferred the option of dropdown boxes instead of textboxes when entering information (e.g., CANRISK scoring). They also had difficulty maneuvering the trackpad and preferred a mouse. Three of them indicated that they were “a little comfortable using computers”, while two said that they were “familiar” with using them. The time to complete data entry per participant ranged between three and thirteen minutes. Table 2 presents the survey results.

**Table 2:** Characteristics and perceptions of users regarding the CP@clinic database.

Domain*	Older adults (set 1) N = 5	Paramedics N = 4	Older adults (set 2) N = 5
Age Group • 20–24 • 25–44 • 45–64 • 65 or older	0 0 5 0	2 2 0 0	0 0 3 2
Familiarity/comfort with using computers • Very comfortable • A little comfortable • Familiar	0 4 1	2 2 0	3 0 2
Difficulty • Easy • Neutral	4 1	4 0	4 1
Time to complete the database • Much quicker than expected • A little quicker than expected • Just right • A little longer than expected	3 2 0 0	2 0 2 0	1 2 1 1
Will paramedics be able to complete the database • Definitely yes	N/A	4	N/A
Will older adult volunteers be able to complete the database • Definitely yes • Probably yes • Probably no	4 1 0	4 0 0	4 0 1

*All responses were 5-point Likert scales except for “Age Group”

#### Pilot test with older adults (set 2)

Pilot testing was then conducted with the second set of older adults, incorporating the suggested changes from the first set. In this refined version of the database, we replaced many of the open test entries to dropdown options, but with an option to enter “others” and an open text space. The results were similar to the first set of participants (Table 2). All participants were able to complete data entry within three and ten minutes. One older adult who reported that completing the database took longer than expected and older adult volunteers would probably not be able to complete the database. Similarly, one participant commented on a specific field in the database that should allow for both text and numerical entries and one participant wanted to improve the response format to a single instead of multiple columns.

#### Pilot test with paramedics

Our final pilot testing was with paramedics who were likely to be the first group to implement the CP@clinic program. All the paramedics found the database easy to use, completed it more quickly than expected, and felt that older adult volunteers and paramedics would be able to complete the data entry. One participant suggested improving the layout to avoid a crowded UI, which was incorporated into our final database.

#### Final database

The final CP@clinic database on the MS Access platform contained 8 pages/forms for data entry (S1). The first four pages were for the initial participant visit, which contained the risk factor assessments. The next page included the participants’ medication history, which was used to monitor if the participants’ medications have been adjusted by their family doctor based on the participant reports (e.g. BP readings). The subsequent page summarized the participants’ risk factors, which lead into the risk factor discussion form. Accordingly, participants were asked to pick one risk factor that they wanted to modify and relevant community resources were then presented as a list. Lifestyle changes made by participants were followed-up in subsequent visits. The final two pages in the database were to be completed during follow-up visits. This included a page for any resources used and health or medication updates since the last visit, as well as a page for the participants’ follow-up BP measurements.

Ongoing maintenance of the database was needed as there was a high turn-over of users (e.g., paramedics) and thus the CP@clinic team had to perform on-going database training/troubleshooting for new paramedics. To assist paramedics, a database guide was created that included screenshots of the database with procedures on properly documenting participant information (S2) as well as a Frequently Asked Questions document (S3). Lastly, an additional interface that allowed easier navigation and search function of participants was added which prevented data entry errors. Overall, these documents were created based on the challenges that arose with new and current paramedics and helped ensure quality and control of the database and participant information.

## Discussion

We have found a low-cost, user-friendly, and secure IT solution that meets our criteria and can be replicated in concept by other healthcare researchers. It captures initial and follow-up data, has built-in algorithms that guide the paramedics in administering the risk assessments, and calculates and summarizes the participants’ risk factor scores based on risk factor tools and other data being collected. The database can provide directions to individuals and information about local resources that is tailored to the participants’ situation. Further, the database provides automated alerts based on specific evidence-based algorithms, generates automated reports, and securely syncs data to a central server. While our paper did not assess risk calculation accuracy, prior studies emphasize the effectiveness of algorithms and tools utilized in programs like CP@clinic. Previous findings include reduced blood pressure, improved quality-adjusted life years (QALYs), and enhanced daily functioning among participants, while the use of tools like CANRISK has uncovered high rates of undiagnosed prediabetes and diabetes in low-income older adults [3,23]. With all database tools validated in prior studies, optimizing the design of the database for user-friendliness, cost-effectiveness, and efficient program delivery is essential.

Recognizing the interaction between healthcare technology and its users is essential for successful implementation, as overlooking this could potentially pose challenges. Previous studies have emphasized the significance of perceived ease of use, perceived usefulness, and security when assessing the adoption of healthcare technology [24,25]. These factors were also highlighted as being important to the end-user in our study during the finalization of the database. Moreover, previous studies have shown that younger healthcare providers and patients demonstrate greater inclination towards using healthcare technologies compared to their older counterparts, who are generally less inclined to engage with healthcare technology [26,27]. It has also previously been reported that low-income older adults experience low health literacy [1]. Given the reduced tendency of older adults to adopt technology along with the limited health literacy of low-income older adults, we chose to test the database with older adult volunteers to ensure its alignment with the specific needs of this demographic. Understanding and addressing the dynamics between healthcare technology and its users are crucial for effectively integrating and utilizing these technologies in healthcare environments.

Next, we will explore the practical aspects of implementing our low-cost computer-based database. The resulting database that was developed required financial outlay only for the hardware and MS Access 2010 or later versions, though MS Access Runtime can be used as a cost-free alternative [17]. A web-based database retains a high upfront cost for set up and maintenance to regularly modify the database and requires a consistent internet connection, which is not feasible in certain locations where CP@clinic sessions were held. In these situations, a database contained within a laptop provides the most flexibility as the data can be saved on the laptop with encryption and synchronized to the central server when internet is available. However, the database user will be required to sync the database as often as possible once internet access is available to ensure participant information is not outdated or lost.

Comparing our database solution to other widely available solutions, such as REDCap or Epi Info, our database can handle the most complex algorithms and data structure. Furthermore, the automated queries allow for data outputs/reports to be readily available. The UI can also be made to be user-friendly with almost unlimited flexibility. The advantage of REDCap and Epi Info is the ease of developing a database, where the front-end and back-end of the database are simultaneously created. Unfortunately, the UI infrastructure is limited, and the offline versions of these databases frequently suffer with data duplication errors upon syncing. Since are goal was to develop a database that could accommodate users with varying levels of experience with technology, the database was changed to MS Access 2010 from Epi Info 7 during pilot testing based on feedback from three paramedics who reported slow data processing speed and a less intuitive UI for the database using Epi Info 7.

In terms of scalability, the Microsoft Access database is easily scalable as the solution requires minimal investment. However, if multiple users on the same database is a desired feature, then a virtual private network or cloud is required for the database to be shared between users. This will require internet connectivity. However, all other solutions requiring seamless access to a database by multiple users will require internet connectivity. The data outputs can also be shared to Electronic Health Records (EHR) as pdf print outs or data tables. However, depending on the EHR, infrastructure to upload the data or an Application Programming Interface (API) needs to be built for this purpose.

Security is an important issue in the collection of electronic patient data and researchers have an ethical duty to ensure that patient data cannot be compromised [28,29]. A standard protocol for data encryption and password protection were set in place. A Yubikey (with a backup copy) was used to store the password and time freeze software was used to prevent malware. Additionally, security was boosted by locking down all applications after one minute if the laptop is unplugged and not in use or when the laptop was in sleep mode, and through anti-theft software that could delete all files remotely if the laptop was registered as stolen.

### Research contributions

This study contributes to both academia and the healthcare sector by introducing a practical solution with versatile applications for similar community healthcare initiatives. The solution, characterized by its cost-effectiveness, portability, security, and user-friendliness, serves as a framework for enhancing healthcare accessibility and efficiency. This contributes to the advancement of patient-centered care practices in the health sector. Healthcare providers can deliver more efficient and effective care, ultimately improving health outcomes for this demographic. Moreover, the study’s findings suggest that the database solution created is scalable and replicable in comparable healthcare environments. The design is adaptable, which can accommodate various contexts and demographics, and the user-friendly nature of the database makes it accessible to a wide range of healthcare providers. The database’s capacity for scalability and adaptability implies that its influence may extend beyond its initial implementation. Academically, this study adds to the body of knowledge regarding effective strategies for managing and supporting older adults in community settings. It also adds to the body of knowledge of a pragmatic process of developing an electronic database with decision support for a community-based primary care program. This research not only addresses a gap between academia and practical healthcare implementation but also serves as a vehicle for improving patient outcomes and enhancing approaches to healthcare delivery in community settings.

### Limitations

These results should be considered in the light of some limitations. The study was limited by the small sample size of participants used to test the database. Although we have variety in the range of prior technological knowledge and skills, this could be further strengthened by increasing the sample size. Moreover, our study focused on one geographical area, and thus it is difficult to generalize the results to all potential program areas. For instance, we did not have volunteers from rural/remote areas, who could have different experiences and perspectives regarding technology, database’s functionality and ease-of-use. Previous research has suggested variations in healthcare technology utilization and skills among healthcare providers and patients between rural and non-rural regions of Canada [26]. This implies a potential avenue for future research into the utilization of the database across different regions of Ontario.

This paper serves as a foundation for future exploration in database development for healthcare settings. There is a need for longitudinal studies to evaluate the impact and efficacy of the developed database solution over a longer period of time, alongside ongoing user feedback to refine it. Furthermore, investigations into the integration of new technologies like artificial intelligence or machine learning would be beneficial as it would provide insight into potential ways to enrich the database’s functionality and effectiveness. Finally, future research may focus on comparative analyses between the developed database solution and existing healthcare administration platforms or chronic disease management tools to reveal the database’s strengths and areas for improvement. By furthering this research, we can ensure that the database solution continues to evolve, maintaining its impact, accessibility, and utility. This, in turn, will improve healthcare delivery and lead to better outcomes for patients.

## Conclusion

The results from the CP@clinic database solution suggest that future paramedics and volunteer older adults that run CP@clinic sessions will find the database easy to use, which can impact the effectiveness and efficiency of the program. The methods used to develop this database are translatable to other health research studies in which ongoing patient data is collected electronically and longitudinally. It presents an inexpensive, reliable, simple and secure method of collecting health data that can be used by researchers and family physicians to improve the health status of patients.

## Supporting information

S1 File User Interface of the CP@clinic database. (PDF)

S2 File Database User Guide. The CP@clinic Database Guide that was created to provide support for current and new paramedics when using the CP@clinic database.
(PDF)

S3 File CP@clinic Database FAQ. The CP@clinic Database FAQ that was created as a supplementary tool for current and new paramedics who may have troubleshooting issues when using the CP@clinic database. Below is a list of common questions that were asked by paramedics.
(PDF)
