# Supplementary material for: Development and evaluation of a low-cost database solution for the Community Paramedicine at Clinic (CP@clinic) database
Source: PLOS Digit Health. 2024 Dec 27;3(12):e0000689. doi: 10.1371/journal.pdig.0000689 (PMC11676497; doi:10.1371/journal.pdig.0000689)
Supplement: S1 File — (PDF) [file pdig.0000689.s001.pdf]

S1 File: User Interface of the CP@clinic database

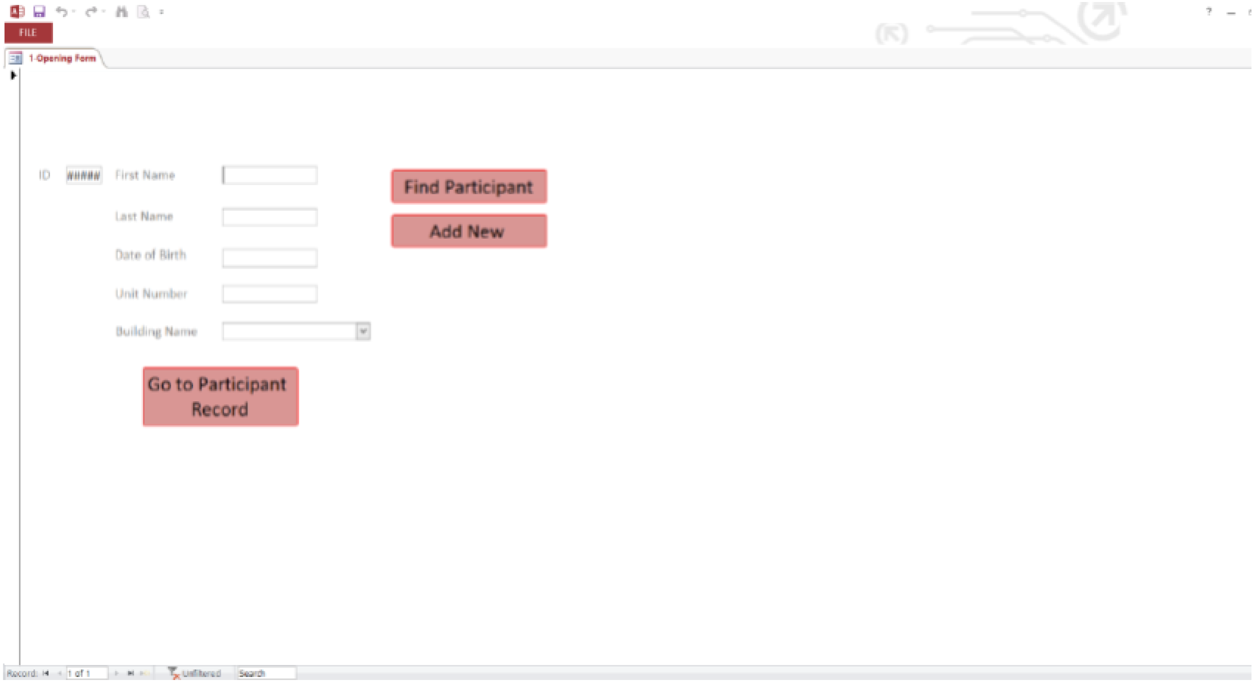

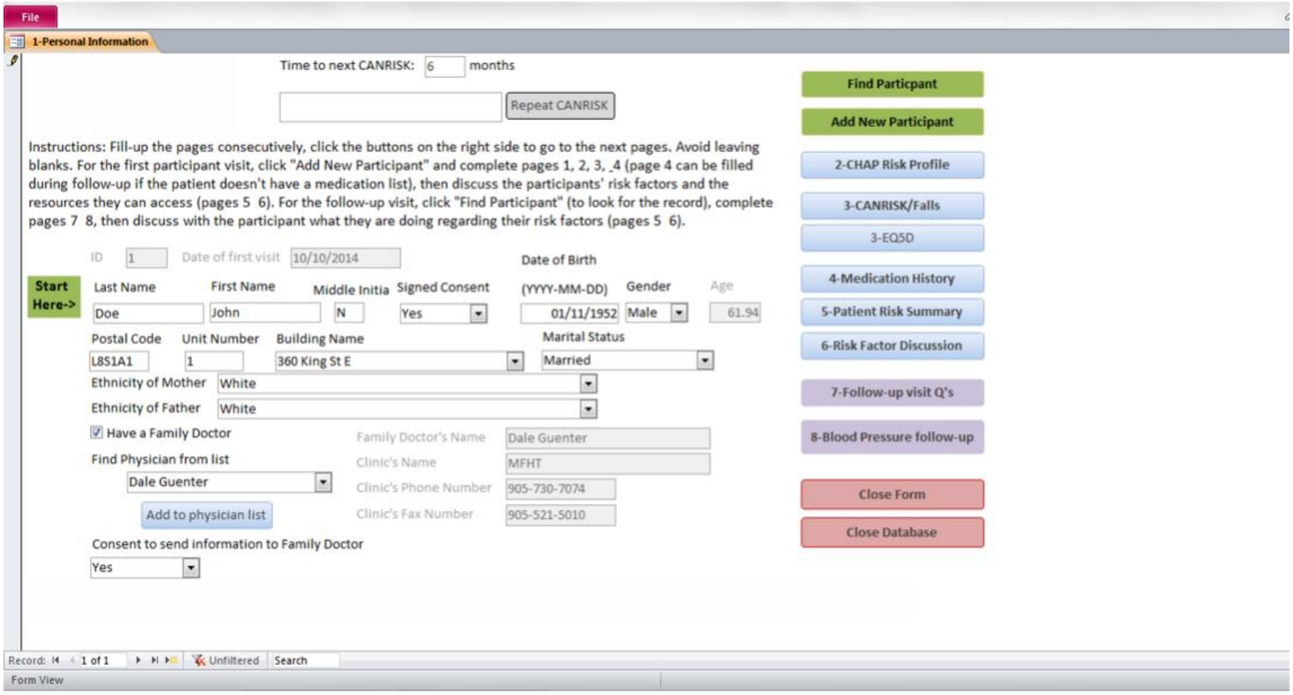

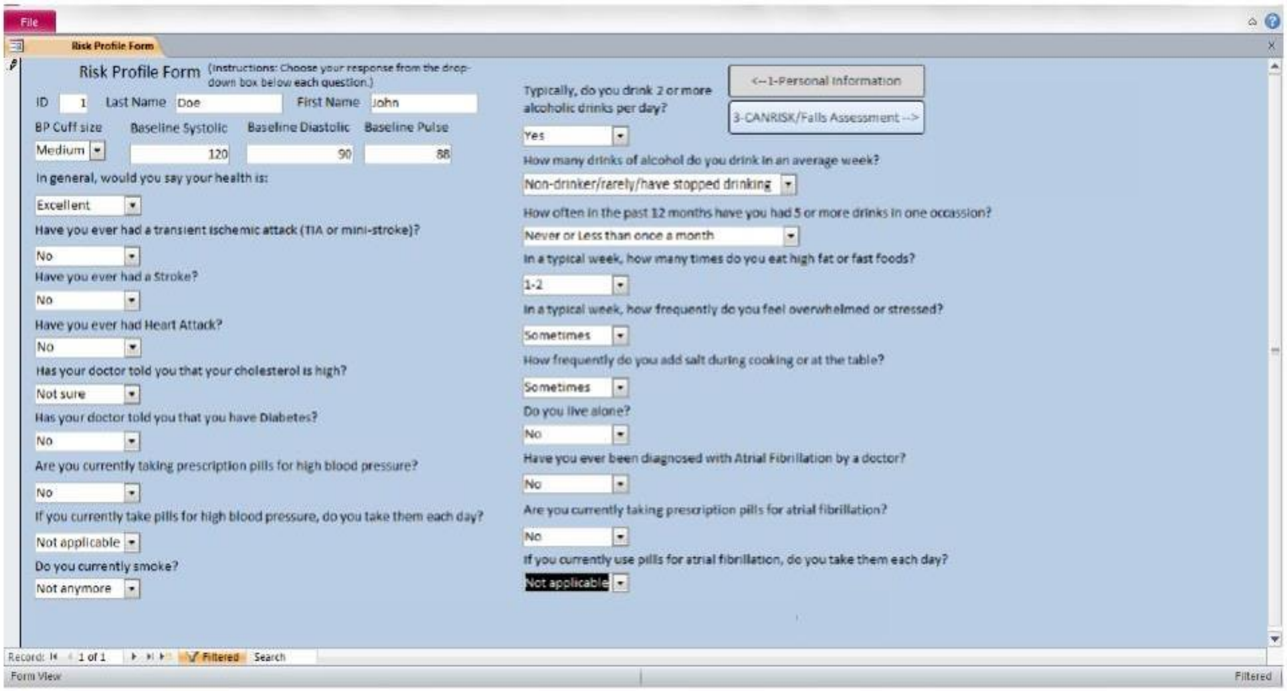

File

Risk Profile Form

Risk Profile Form (Instructions: Choose your response from the drop-down box below each question.)

ID 1 Last Name Doe First Name John

BP Cuff size: Medium | Baseline Systolic: 120 | Baseline Diastolic: 90 | Baseline Pulse: 88

In general, would you say your health is: Excellent

Have you ever had a transient ischemic attack (TIA or mini-stroke)? No

Have you ever had a Stroke? No

Have you ever had Heart Attack? No

Has your doctor told you that your cholesterol is high? Not sure

Has your doctor told you that you have Diabetes? No

Are you currently taking prescription pills for high blood pressure? No

If you currently take pills for high blood pressure, do you take them each day? Not applicable

Do you currently smoke? Not anymore

Typically, do you drink 2 or more alcoholic drinks per day? Yes

<--1-Personal Information

3-CANRISK/Falls Assessment -->

How many drinks of alcohol do you drink in an average week? Non-drinker/rarely/have stopped drinking

How often in the past 12 months have you had 5 or more drinks in one occassion? Never or Less than once a month

In a typical week, how many times do you eat high fat or fast foods? 1-2

In a typical week, how frequently do you feel overwhelmed or stressed? Sometimes

How frequently do you add salt during cooking or at the table? Sometimes

Do you live alone? No

Have you ever been diagnosed with Atrial Fibrillation by a doctor? No

Are you currently taking prescription pills for atrial fibrillation? No

If you currently use pills for atrial fibrillation, do you take them each day? Not applicable

Record: 1 of 1 Filtered Search

Form View Filtered

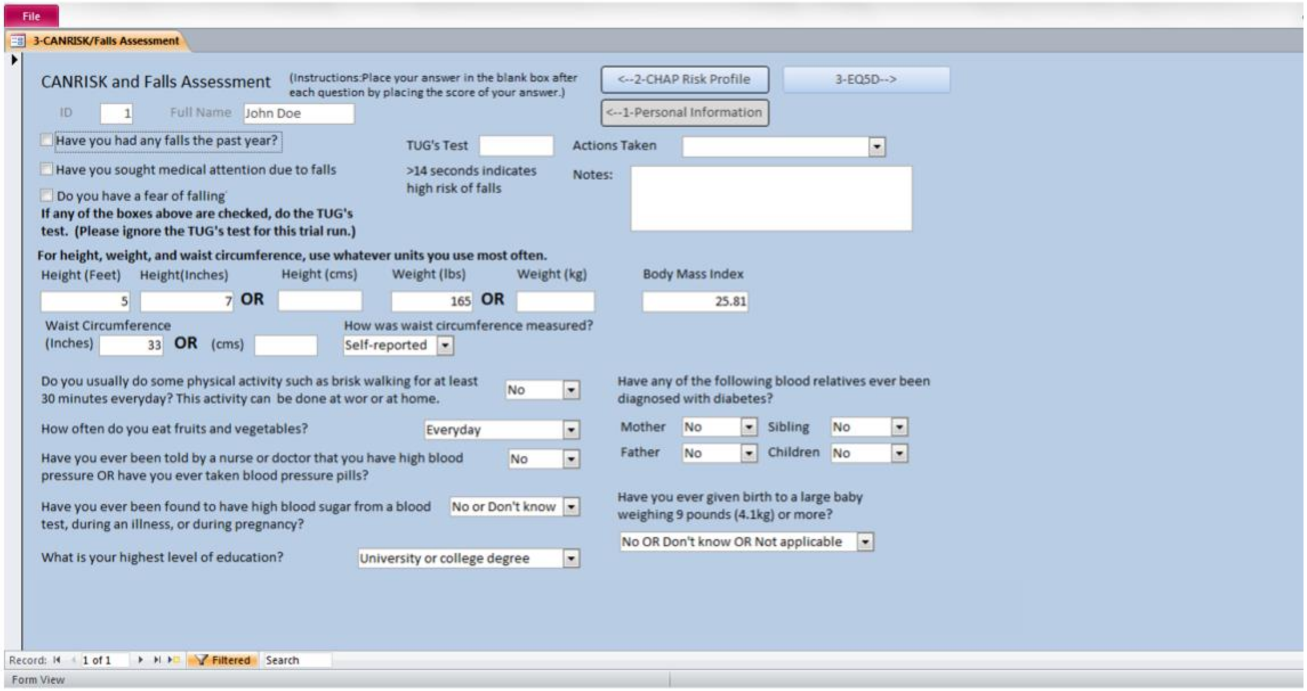

File

3-CANRISK/Falls Assessment

CANRISK and Falls Assessment (Instructions:Place your answer in the blank box after each question by placing the score of your answer.)

<--2-CHAP Risk Profile | 3-EQ5D-->

<--1-Personal Information

ID 1 Full Name John Doe

Have you had any falls the past year?

Have you sought medical attention due to falls

Do you have a fear of falling

If any of the boxes above are checked, do the TUG's test. (Please ignore the TUG's test for this trial run.)

TUG's Test

>14 seconds indicates high risk of falls

Actions Taken

Notes:

For height, weight, and waist circumference, use whatever units you use most often.

Height (Feet)	Height(Inches)		Height (cms)	Weight (lbs)		Weight (kg)	Body Mass Index
5	7	OR		165	OR		25.81

Waist Circumference (Inches) 33 OR (cms)

How was waist circumference measured? Self-reported

Do you usually do some physical activity such as brisk walking for at least 30 minutes everyday? This activity can be done at wor or at home. No

How often do you eat fruits and vegetables? Everyday

Have you ever been told by a nurse or doctor that you have high blood pressure OR have you ever taken blood pressure pills? No

Have you ever been found to have high blood sugar from a blood test, during an illness, or during pregnancy? No or Don't know

What is your highest level of education? University or college degree

Have any of the following blood relatives ever been diagnosed with diabetes?

Mother No | Sibling No

Father No | Children No

Have you ever given birth to a large baby weighing 9 pounds (4.1kg) or more? No OR Don't know OR Not applicable

Record: 1 of 1 Filtered Search

Form View

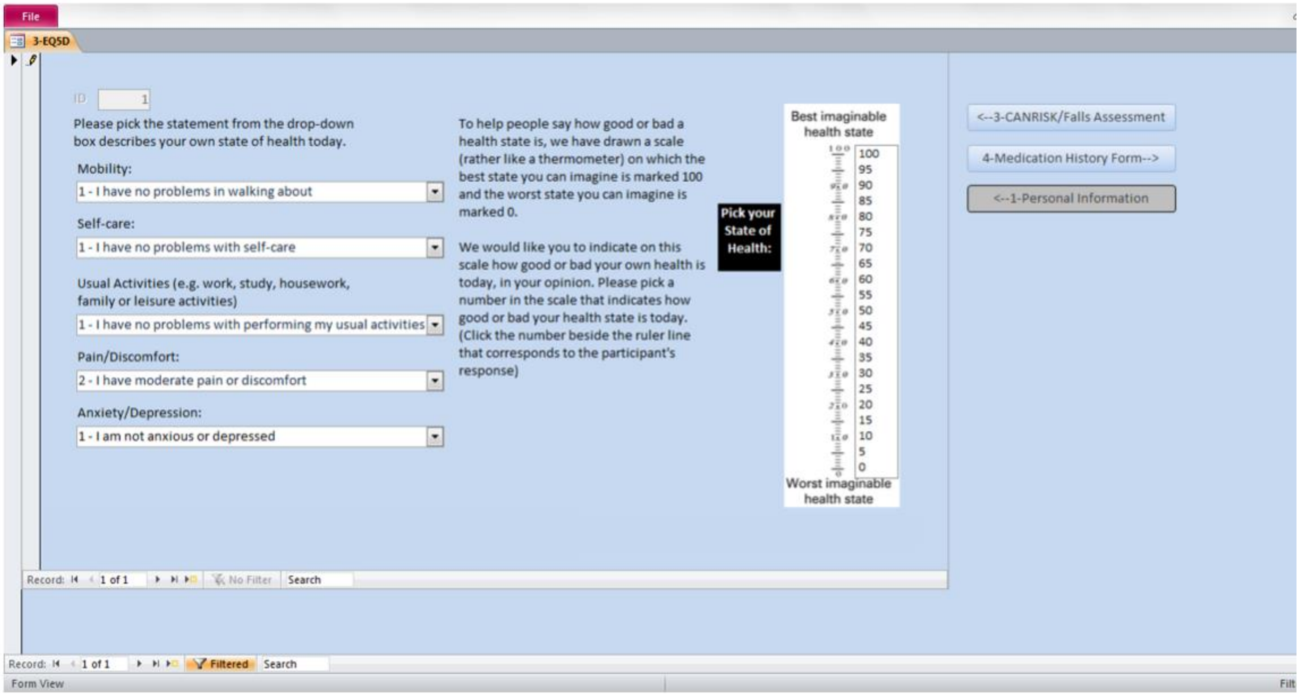
File
3-EQ5D
ID 1
Please pick the statement from the drop-down box describes your own state of health today.
Mobility:
1 - I have no problems in walking about
Self-care:
1 - I have no problems with self-care
Usual Activities (e.g. work, study, housework, family or leisure activities)
1 - I have no problems with performing my usual activities
Pain/Discomfort:
2 - I have moderate pain or discomfort
Anxiety/Depression:
1 - I am not anxious or depressed
To help people say how good or bad a health state is, we have drawn a scale (rather like a thermometer) on which the best state you can imagine is marked 100 and the worst state you can imagine is marked 0.
We would like you to indicate on this scale how good or bad your own health is today, in your opinion. Please pick a number in the scale that indicates how good or bad your health state is today. (Click the number beside the ruler line that corresponds to the participant's response)
Pick your State of Health:
Best imaginable health state
100
95
90
85
80
75
70
65
60
55
50
45
40
35
30
25
20
15
10
5
0
Worst imaginable health state
<--3-CANRISK/Falls Assessment
4-Medication History Form-->
<--1-Personal Information
Record: 1 of 1 No Filter Search
Record: 1 of 1 Filtered Search
Form View

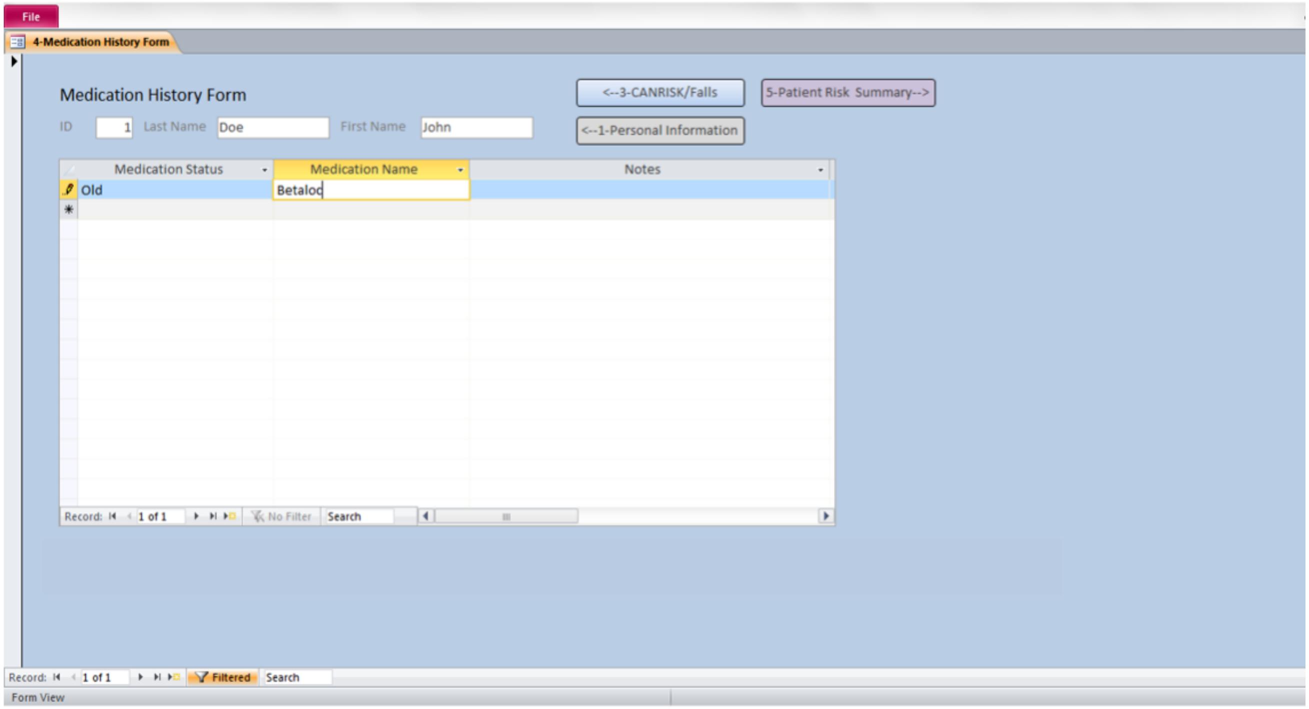
File
4-Medication History Form
Medication History Form
ID 1 Last Name Doe First Name John
<--3-CANRISK/Falls
5-Patient Risk Summary-->
<--1-Personal Information
Medication Status
Medication Name
Notes
Old
Betaloc
Record: 1 of 1 No Filter Search
Record: 1 of 1 Filtered Search
Form View

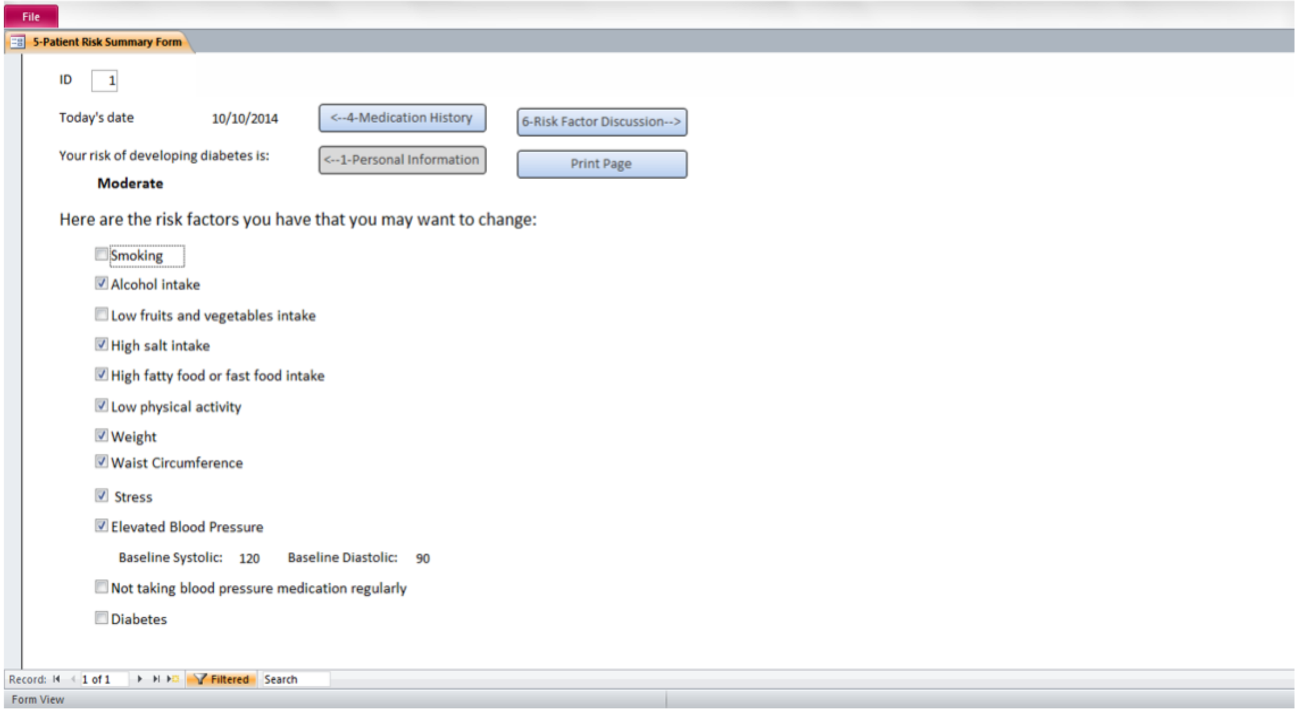
File
5-Patient Risk Summary Form
ID 1
Today's date 10/10/2014
<--4-Medication History
6-Risk Factor Discussion-->
Your risk of developing diabetes is:
<--1-Personal Information
Print Page
Moderate
Here are the risk factors you have that you may want to change:
Smoking
Alcohol intake
Low fruits and vegetables intake
High salt intake
High fatty food or fast food intake
Low physical activity
Weight
Waist Circumference
Stress
Elevated Blood Pressure
Baseline Systolic: 120 Baseline Diastolic: 90
Not taking blood pressure medication regularly
Diabetes
Record: 1 of 1 Filtered Search
Form View

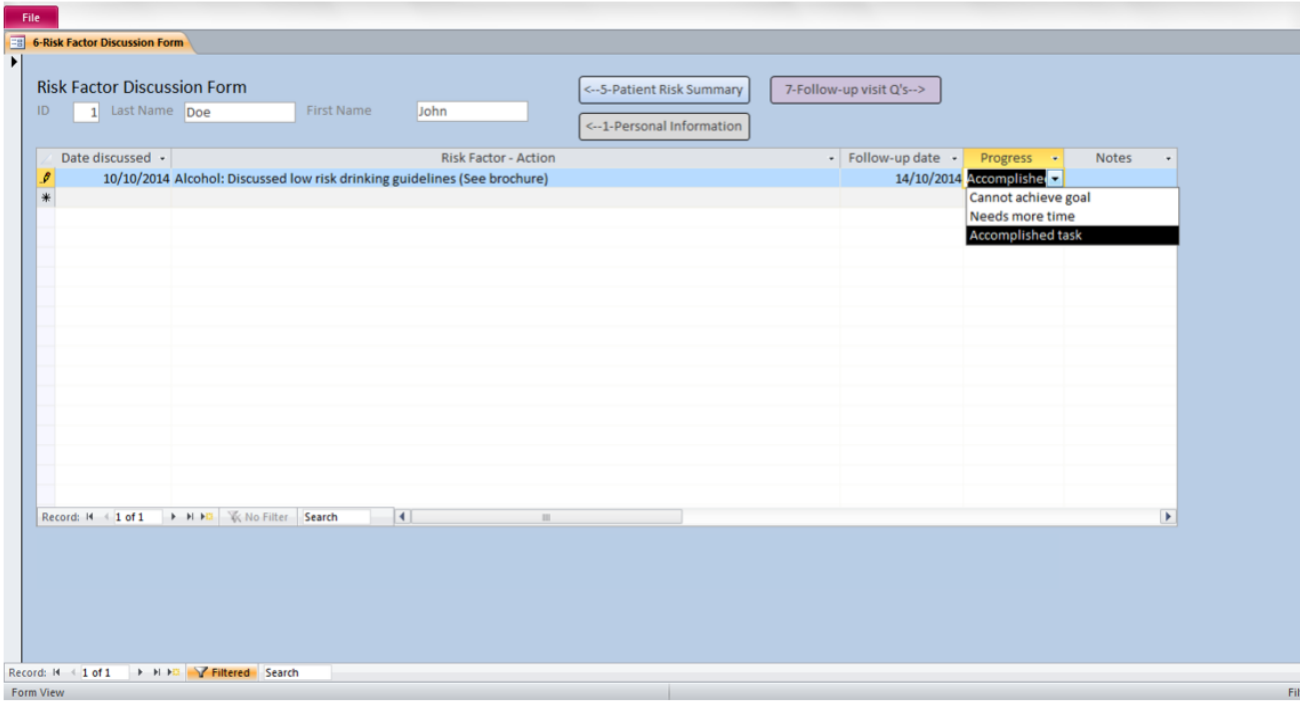
File
6-Risk Factor Discussion Form
Risk Factor Discussion Form
<--5-Patient Risk Summary
7-Follow-up visit Q's-->
ID 1 Last Name Doe First Name John
<--1-Personal Information
Date discussed
Risk Factor - Action
Follow-up date
Progress
Notes
10/10/2014 Alcohol: Discussed low risk drinking guidelines (See brochure)
14/10/2014
Cannot achieve goal
Needs more time
Accomplished task
Record: 1 of 1 No Filter Search
Record: 1 of 1 Filtered Search
Form View

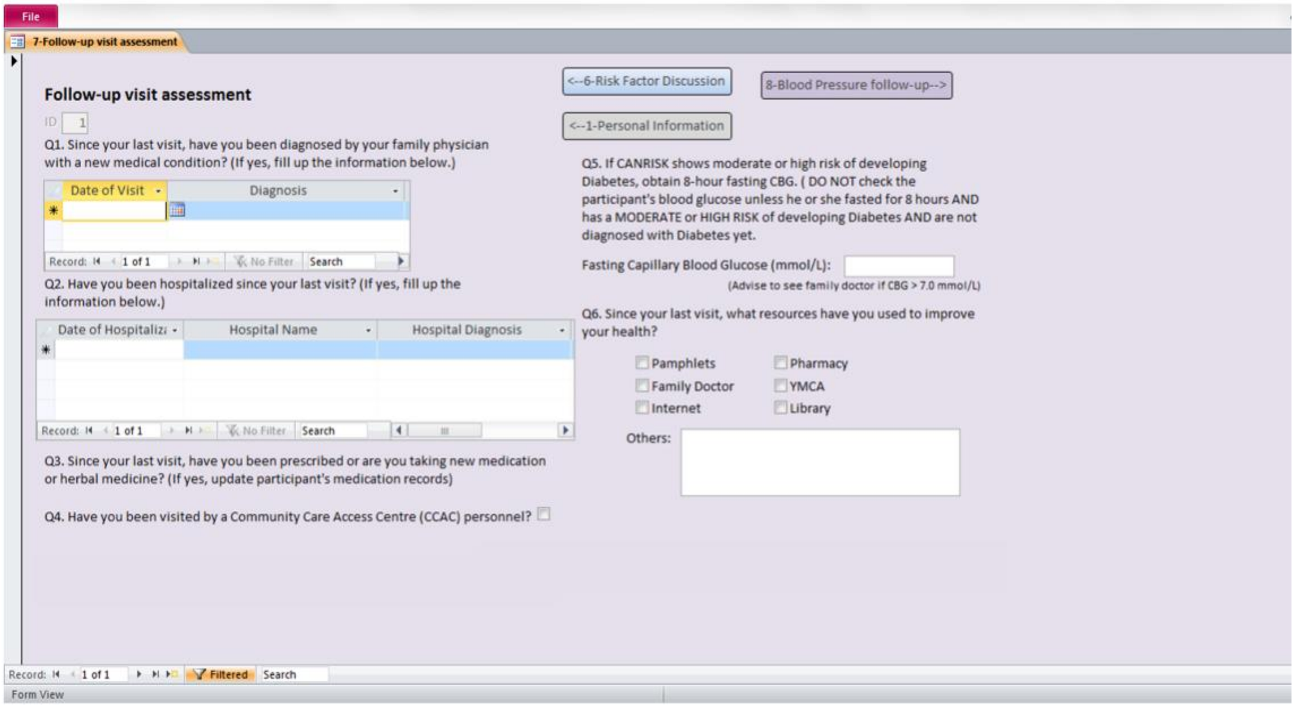

File

7-Follow-up visit assessment

Follow-up visit assessment

ID 1

Q1. Since your last visit, have you been diagnosed by your family physician with a new medical condition? (If yes, fill up the information below.)

Date of Visit	Diagnosis

Record: 1 of 1 No Filter Search

Q2. Have you been hospitalized since your last visit? (If yes, fill up the information below.)

Date of Hospitaliz:	Hospital Name	Hospital Diagnosis

Record: 1 of 1 No Filter Search

Q3. Since your last visit, have you been prescribed or are you taking new medication or herbal medicine? (If yes, update participant's medication records)

Q4. Have you been visited by a Community Care Access Centre (CCAC) personnel? ☐

<--6-Risk Factor Discussion

8-Blood Pressure follow-up-->

<--1-Personal Information

Q5. If CANRISK shows moderate or high risk of developing Diabetes, obtain 8-hour fasting CBG. (DO NOT check the participant's blood glucose unless he or she fasted for 8 hours AND has a MODERATE or HIGH RISK of developing Diabetes AND are not diagnosed with Diabetes yet.

Fasting Capillary Blood Glucose (mmol/L):

(Advise to see family doctor if CBG > 7.0 mmol/L)

Q6. Since your last visit, what resources have you used to improve your health?

- ☐ Pamphlets
- ☐ Pharmacy
- ☐ Family Doctor
- ☐ YMCA
- ☐ Internet
- ☐ Library

Others:

Record: 1 of 1 Filtered Search

Form View

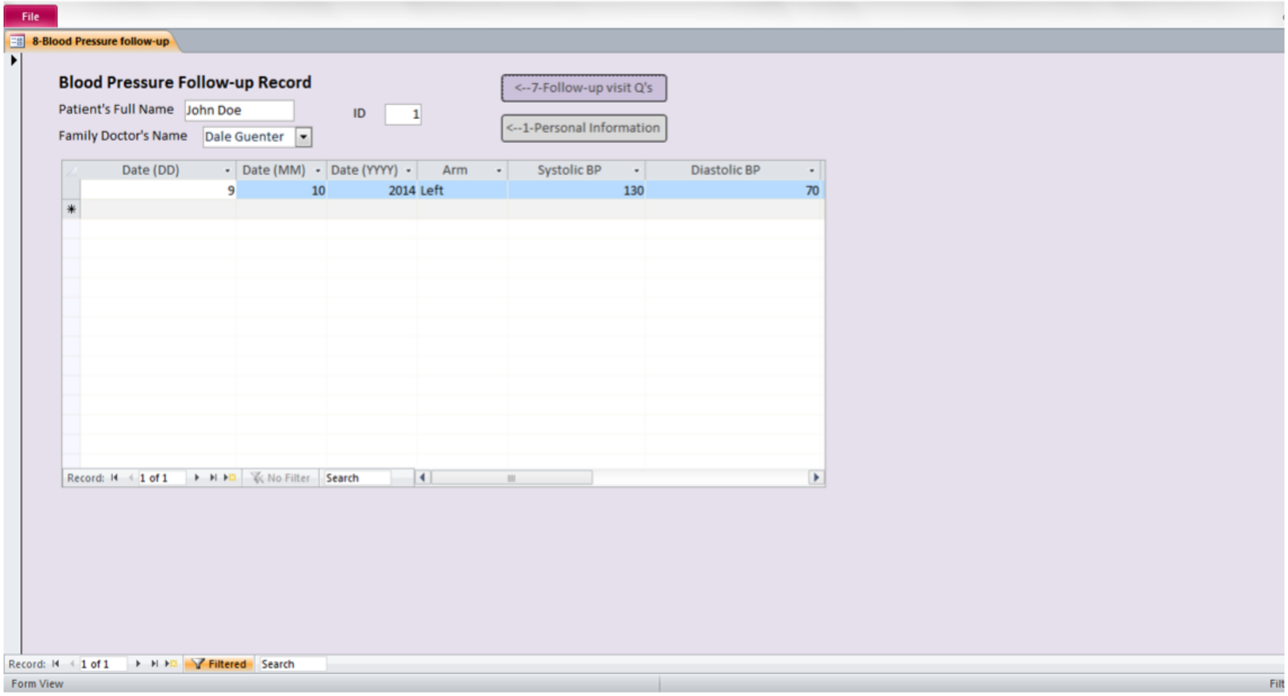

File

8-Blood Pressure follow-up

Blood Pressure Follow-up Record

Patient's Full Name John Doe

Family Doctor's Name Dale Guenter

ID 1

<--7-Follow-up visit Q's

<--1-Personal Information

Date (DD)	Date (MM)	Date (YYYY)	Arm	Systolic BP	Diastolic BP
9	10	2014	Left	130	70

Record: 1 of 1 No Filter Search

Record: 1 of 1 Filtered Search

Form View
