# Supplementary material for: Development and evaluation of a low-cost database solution for the Community Paramedicine at Clinic (CP@clinic) database
Source: PLOS Digit Health. 2024 Dec 27;3(12):e0000689. doi: 10.1371/journal.pdig.0000689 (PMC11676497; doi:10.1371/journal.pdig.0000689)
Supplement: S2 File — The CP@clinic Database Guide that was created to provide support for current and new paramedics when using the CP@clinic database. (PDF) [file pdig.0000689.s002.pdf]

S2 File: Database User Guide

The CP@clinic Database Guide that was created to provide support for current and new paramedics when using the CP@clinic database.

CP@clinic Database Guide

Introduction

Welcome to the CP@clinic program. This is the CP@clinic Database Guide designed to help all current and future users of the CP@clinic database. This is a detailed easy step-by-step guide that provides instructions from inputting the participants' initial visit information to follow-up, as well as syncing the database to the McMaster server. This guide includes screenshots and highlights features and functions of the database. It is designed to follow the flow as you progress to capture the participant's information during the CP@clinic sessions. **A sample (practice) database has also been created and is located in a folder on the desktop of the computer for current and new users to practice data entry and test run the database. Please note that actual participant information should NOT be stored on the sample database and please do not use the actual database to practice data entry.**

If you have any questions, please contact feel free to contact us with the coordinates provided below.

Ric Angeles, Research Associate
Tele: (289)489-1337
Email: angelesric@gmail.com

Annie Lok, Research Coordinator
Tele: (905) 525-9140 Ext. 21217
Email: lokaym@mcmaster.ca
