# Supplementary material for: Development and evaluation of a low-cost database solution for the Community Paramedicine at Clinic (CP@clinic) database
Source: PLOS Digit Health. 2024 Dec 27;3(12):e0000689. doi: 10.1371/journal.pdig.0000689 (PMC11676497; doi:10.1371/journal.pdig.0000689)
Supplement: S3 File — The CP@clinic Database FAQ that was created as a supplementary tool for current and new paramedics who may have troubleshooting issues when using the CP@clinic database. Below is a list of common questions that were asked by paramedics. (PDF) [file pdig.0000689.s003.pdf]

S3 File: CP@clinic Database FAQ

The CP@clinic Database FAQ that was created as a supplementary tool for current and new paramedics who may have troubleshooting issues when using the CP@clinic database. Below is a list of common questions that were asked by paramedics.

Question 1: How do I open the database where I input the participant's information?

To open the database, you will need the password to the project-designated computer and the encrypted password in the USB key device. If you are still having difficulty, please refer to the Database User Guide for a detailed step-to-step process on accessing the database.

Question 2: How do I add notes about the participant?

There are two approaches to added notes about the participant. If the paramedic wants to note any key concerns that can be further discussed in the next CP@clinic session, the *1-Personal Information* page has a reminder section on the page that allows you to input notes.

Paramedics can also make notes regarding the participant's health during their risk factor discussion portion and can also be added in the **6-Risk Factor Discussion Form** notes column.

Question 3: How do I save the participant information?

As you progress through each form during the health assessment, the information automatically saves. As a safe measure we have included a Save Participant Information button on the main 1-Personal Information page where you can click and the information will be saved on the database. For more detailed instructions on saving information on the database, please refer to the Database User Guide.

Question 4: How do I add additional resources to the database?

If you notice there are resources you want to add that were not captured in the database, please contact the CP@clinic team. All resources on the database have been reviewed and provided by Public Health. The CP@clinic program does not solicit specific resources particularly from for-profit organizations.

Question 6: How do I make changes or update the participant information if I input it incorrectly?

Paramedics can review and update the participant information on an ongoing basis at every CP@clinic session. However, depending on the information that needs to be changed (E.g. accidentally deleted a participant), the paramedic must contact the CP@clinic research team immediately.
